# Perioperative propranolol during robotic‐assisted radical prostatectomy: A randomised, double‐blind, placebo‐controlled, pilot trial (PeP‐RALP)

**DOI:** 10.1002/bco2.70267

**Published:** 2026-08-26

**Authors:** Shivanthe Sivanesan, Håkon Ramberg, Thomas Kristiansen, Suraj B. Thapa, Marthe Bjøre, Viktor Berge, Fredrik Ottosson, Bjørn Brennhovd, Anne M. H. Storås, Per M. Thorsby, Sandra R. Dahl, Alfonso Urbanucci, Helene Hartvedt Grytli, Lars Bjerke, Kristin Austlid Taskén

**Affiliations:** ^1^ Department of Cancer Surgery The Norwegian Radium Hospital, Oslo University Hospital Oslo Norway; ^2^ Department of Tumor Biology, Institute of Cancer Research Oslo University Hospital Oslo Norway; ^3^ Institute of Clinical Medicine University of Oslo Oslo Norway; ^4^ Department Anaesthesia, Operations and Intensive Care The Norwegian Radium Hospital, Oslo University Hospital Oslo Norway; ^5^ Division of Mental Health and Addiction Oslo University Hospital Oslo Norway; ^6^ Department of Oncology The Norwegian Radium Hospital, Oslo University Hospital Oslo Norway; ^7^ Hormone Laboratory, Department of Medical Biochemistry and Endocrinology and Metabolism Research Group Oslo University Hospital Oslo Norway; ^8^ Center for Cancer Eradication Research, Faculty of Medicine and Health Technology Tampere University and TAYS Cancer Center, Tampere University Hospital Tampere Finland; ^9^ Finnish Cancer Institute Helsinki Finland; ^10^ Norsk legemiddelhåndbok Oslo Norway; ^11^ Prostatakreftforeningen (PROFO) Oslo Norway

**Keywords:** feasibility trial, perioperative propranolol, prostate cancer, radical prostatectomy, surgical stress

## Abstract

**Objective:**

This study aimed to determine the feasibility and safety of administering perioperative propranolol, as an adjunct to reduce prostate cancer recurrence, in patients undergoing robotic‐assisted laparoscopic prostatectomy (RALP) for European Association of Urology (EAU) intermediate‐ or high‐risk prostate cancer.

**Patients and methods:**

Treatment‐naïve men aged 40–80 years with EAU intermediate‐ or high‐risk prostate cancer scheduled for RALP were enrolled in this single‐centre, randomised, double‐blind, placebo‐controlled, phase 2 pilot trial. Participants were randomised 1:1 to receive propranolol or placebo for approximately 3 weeks (about 1 week pre‐RALP and 2 weeks post‐RALP). Serial blood sampling was performed to measure catecholamine metabolites, serum propranolol and short‐interval prostate‐specific antigen (PSA) dynamics. Participants self‐monitored pulse rate and blood pressure twice daily.

**Results:**

Over 36 weeks in 2023, 160 patients underwent RALP; 111 were prescreened, 49 were fully screened and 40 were randomised, corresponding to inclusion rates of 36% among prescreened and 82% among fully screened patients. Pre‐RALP adherence was 98% in both arms; post‐RALP adherence was lower in the propranolol arm. No Common Terminology Criteria for Adverse Events (CTCAE) version 5.0 grade ≥3 adverse events were attributed to propranolol, and no surgeries were delayed. The epinephrine metabolite metanephrine increased intraoperatively in both arms and declined by the end of treatment. Mean preoperative PSA decreased more with propranolol than with placebo. As this pilot was not powered to detect between‐group differences, such differences are hypothesis‐generating only.

**Conclusions:**

PeP‐RALP demonstrated that perioperative propranolol is operationally feasible and demonstrated an acceptable safety profile within RALP workflows, with strong preoperative adherence. Our study supports a multicentre efficacy randomised controlled trial with standardised anaesthetic protocols, postoperative adherence support and oncological endpoints. All physiological and molecular results, including PSA dynamics, in this pilot are exploratory and solely intended to inform the design of any future trials.

## 1 INTRODUCTION

Robotic‐assisted laparoscopic prostatectomy (RALP) is a cornerstone of curative treatment for prostate cancer. Nevertheless, a substantial proportion of men with European Association of Urology (EAU) intermediate‐ and high‐risk prostate cancer develop biochemical recurrence (BCR) after radical prostatectomy, underscoring the need for additional strategies to reduce recurrence risk.

Paradoxically, radical surgery with curative intent, including RALP, may promote cancer progression. The perioperative period represents a biologically dynamic window that can influence long‐term oncological outcomes.^1^, ^2^, ^3^, ^4^ Surgical and psychological stress acutely elevates catecholamines, activating β_2_‐adrenergic receptor signalling that can remodel tumour–host compartments (cancer cell plasticity, angiogenesis, immune tone and stromal–vascular interactions), thereby facilitating metastatic seeding and growth of residual disease.^2^, ^3^, ^5^, ^6^, ^7^, ^8^, ^9^

Accumulating translational and clinical evidence suggests that perioperative β*‐*adrenergic blockade may mitigate these pro‐metastatic effects and act as an adjunct to surgery. Randomised phase 2 trials in breast and colorectal cancer demonstrate that propranolol—alone or together with cyclooxygenase‐2 (COX‐2) inhibition—can safely reduce pro‐metastatic biomarkers and modulate immune and inflammatory signatures associated with poor prognosis.^7^, ^10^, ^11^, ^12^ Complementing this, our group has previously shown in a pharmaco‐epidemiologic registry study that incidental non‐selective β‐blocker use at the time of radical prostatectomy was associated with a reduced need for subsequent treatment for recurrence.^13^

Although a randomised controlled trial (RCT) is required to assess oncological efficacy, a feasibility study was warranted to address uncertainties regarding patient recruitment challenges experienced in similar trials,^14^, ^15^ perioperative safety and workflow impact, such as delayed surgical scheduling.

Hence, we conducted the pilot PeP‐RALP, a double‐blind, placebo‐controlled, randomised pilot trial to assess the feasibility and safety of perioperative propranolol versus matched placebo in men undergoing RALP for EAU intermediate‐ and high‐risk prostate cancer, with secondary physiological and molecular endpoints.

## 2 PATIENTS AND METHODS

### 2.1 Ethics and patient involvement

The study adhered to the Declaration of Helsinki, International Council for Harmonisation Good Clinical Practice (ICH‐GCP) and CONSORT (Consolidated Standards of Reporting Trials) guidelines. Approvals and registrations were obtained before patient enrolment (EudraCT 2022‐001184‐28, registered 6 June 2022; ClinicalTrials.gov NCT05679193, registered 12 December 2022; Norwegian Medicines Agency 22/14389‐9; Regional Committees for Medical and Health Research Ethics 488466).

The current protocol (version 3.0; 19 March 2023) was in effect during recruitment. A summary of protocol amendments is provided in the Supporting Information (Protocol amendment history). Key clarifications included refinement of exclusion criteria, subdivision of concomitant medications and adjustments to dose‐modification criteria and safety‐reporting procedures.

Written informed consent was obtained from all participants prior to any study‐specific procedure.

The Norwegian Prostate Cancer Patient Association was engaged during protocol development, and a representative (L. Bjerke) contributed to refining study logistics (timing of study visits) and to improving the clarity and accessibility of the informed consent form for participants.

### 2.2 PeP‐RALP design and setting

**FIGURE 1:**
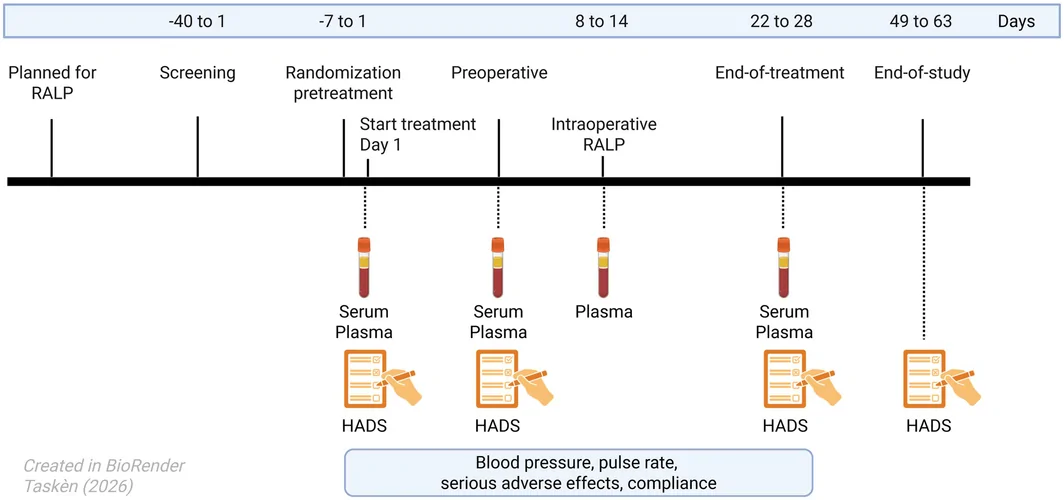
Overview of the PeP‐RALP study. Men planned for robotic‐assisted laparoscopic prostatectomy (RALP) were screened (Days −40 to 1). Eligible participants were randomised 1:1 to receive propranolol or placebo for approximately 1 week preoperatively (Days 1–7), with study medication continued through the perioperative and early postoperative period (Days 8–22/28). End‐of‐treatment assessments were performed on Days 22–28, and an end‐of‐study visit was performed on Days 49–63. Serum and plasma samples were obtained at the start of treatment, preoperatively, intraoperatively (during RALP) and at the end of treatment; Hospital Anxiety and Depression Scale (HADS) questionnaires were collected at the start of treatment, preoperatively, at the end of treatment and at the end of study. Throughout the dosing period, participants monitored blood pressure and pulse rate and recorded serious adverse events and treatment compliance.

PeP‐RALP was a double‐blind, placebo‐controlled, randomised, phase 2 trial designed to assess the feasibility of a future efficacy trial comparing perioperative propranolol versus matched placebo to attenuate perioperative stress‐induced cancer progression in men undergoing RALP for EAU intermediate‐ and high‐risk prostate cancer (Figure 1).

The start of the study (first screening) was 04.01.2023; first/last enrolment was 10.01.2023/05.12.2023; last‐patient follow‐up was 26.01.2024; the medical monitoring end‐of‐study evaluation was 23.04.2024; database lock was 24.05.2024; and analyses were completed on 16.12.2025.

### 2.3 Participants

Eligible participants were treatment‐naïve men aged 40–80 years with EAU intermediate‐ and high‐risk prostate cancer, without known distant metastasis (M0/Mx) and with an Eastern Cooperative Oncology Group Performance Status (ECOG PS) of 0–1, scheduled for RALP at our hospital, and able and willing to provide written informed consent in Norwegian.

Key exclusion criteria were significant cardiopulmonary disease (e.g., unstable angina and decompensated heart failure), asthma/bronchospasm, atrioventricular block ≥2, baseline bradycardia (<60 beats per minute [bpm]) or hypotension (<110/60 mmHg), and systemic β‐blocker use within the last 3 months. A full list of inclusion and exclusion criteria is found in Table S1.

### 2.4 Randomisation and masking

Participants were randomised 1:1 using an interactive web response system (Viedoc©), with allocation concealment by centralised dispensing. Participants, clinical teams and assessors were blinded.

Study medication was prepared and packaged by an external manufacturer (Kragerø Tablettproduksjon AS); capsules and containers for both arms were identical in appearance.

### 2.5 Overview of study interventions and procedures

Dosing consisted of 20 mg twice daily on Days 1–3; 40 mg twice daily on Days 4–19; and 20 mg twice daily on Days 20–22; an extension of up to 6 days was permitted. Escalation on Day 4 required pulse rate ≥60 bpm and blood pressure (BP) ≥110/60 mmHg.

For home monitoring, participants recorded twice‐daily pulse rate and BP, study‐medication intake (compliance) and adverse events (AEs).

Blood sampling and Hospital Anxiety and Depression Scale (HADS) assessments were conducted as shown in Figure 1; further details are provided in the Schedule of Activities (Supporting Information).

Intraoperatively, non‐invasive cerebral blood flow velocity was assessed using transcranial Doppler (TCD) through the temporal window,^16^ post‐induction (supine and pre‐pneumoperitoneum) and during vesicourethral anastomosis (30° Trendelenburg and pneumoperitoneum), contingent on operator availability.

### 2.6 Outcomes

#### 2.6.1 Primary

1. Screening‐to‐enrolment ratio to achieve the planned sample size.
2. Medication adherence, defined as >80% intake of planned doses, preoperatively and postoperatively.

#### 2.6.2 Secondary

1. Safety/tolerability: AEs (patient‐reported and investigator‐assessed) graded per Common Terminology Criteria for Adverse Events (CTCAE) v5.0; home and perioperative haemodynamics; intraoperative challenges; and delays and cancellations of RALP due to study participation.
2. Intraoperative parameters: Anaesthesia duration, procedure time, blood loss and use of vasoactive medication.
3. Catecholamine dynamics: Perioperative changes in plasma metanephrine and normetanephrine.
4. Short‐interval prostate‐specific antigen (PSA) changes: Changes in serum PSA after 7–14 days of preoperative propranolol or placebo.
5. Bioavailability of oral propranolol: Serial measures of serum propranolol concentrations.

#### 2.6.3 Exploratory

Alterations in perceived distress (HADS) and cerebral blood flow velocity (TCD) were assessed.

A full list of the objectives and endpoints is found in Table S2.

### 2.7 Safety assessments and reporting

AEs and serious AEs (SAEs) were collected from first study dose through end of follow‐up and reported per regulatory requirements; an independent medical monitor periodically reviewed safety.

### 2.8 Statistical analysis

This pilot study (planned *N* = 40) was designed to generate feasibility and safety estimates to inform the design and power calculations of a future efficacy RCT. The sample size reflected the number of participants expected to be included over 6 months by screening 150–200 patients. Drug compliance was summarised per treatment arm to estimate expected adherence rates. Safety analyses included participants who received at least one dose of study medication.

No formal hypothesis testing was prespecified to detect between‐group differences. Analyses were descriptive with continuous variables summarised as mean (SD) and categorical variables as number (*N*; %). The software used was R/R‐Studio^17^ and IBM SPSS v30.

## 3 RESULTS

### 3.1 Participant recruitment and baseline characteristics

**FIGURE 2:**
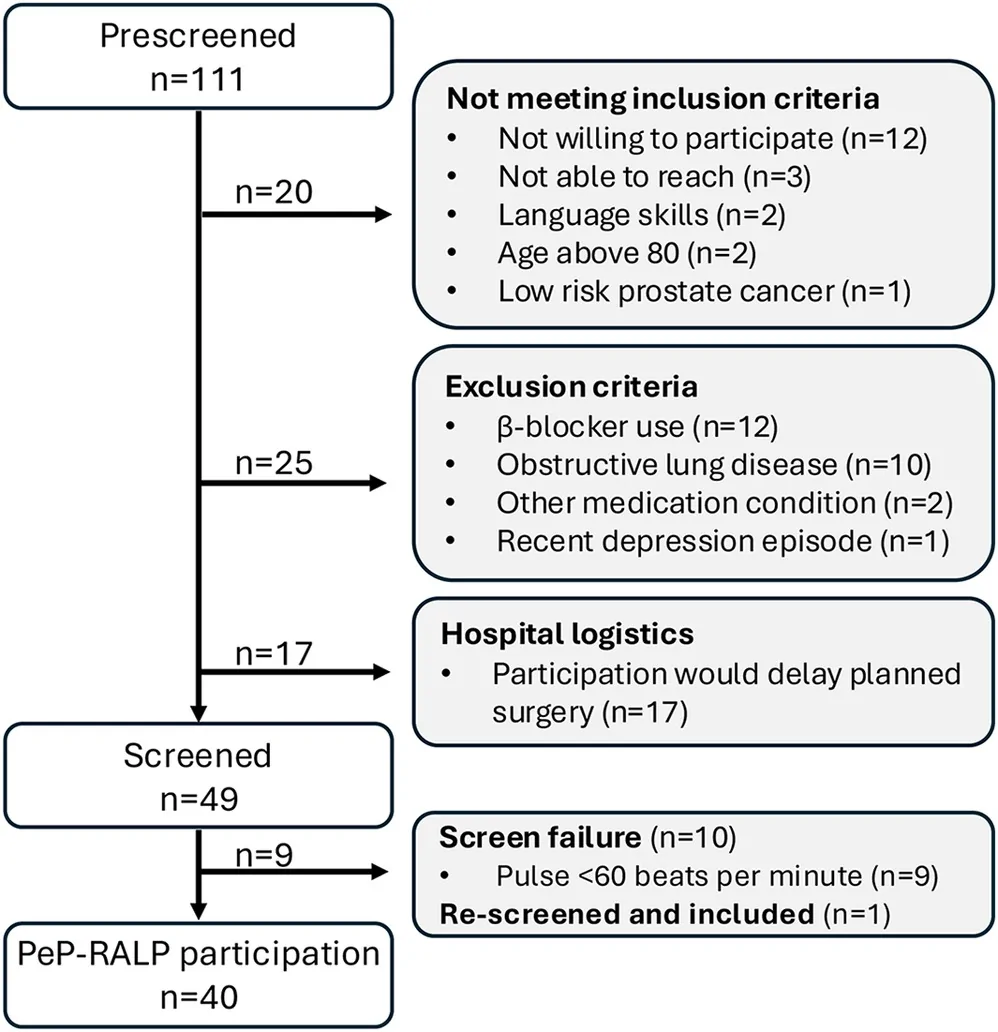
Participant flow; recruitment and exclusions. Over a 36‐week screening period in 2023 (Batch 1: Weeks 1–22; Batch 2: Weeks 34–47), 111 patients scheduled for robotic‐assisted laparoscopic prostatectomy (RALP) were identified and prescreened. Ninety‐one met the inclusion criteria, and 25 were excluded due to prespecified exclusion criteria identified at prescreening; a further 17 otherwise eligible patients were not included because study participation would have delayed planned surgery. Of 49 fully screened patients, 40 were enrolled and randomised 1:1 to receive perioperative propranolol or placebo, forming the study cohort for analysis.

Over 36 weeks, 160 patients underwent RALP; 111 were prescreened, 49 were fully screened and 40 were randomised 1:1 to propranolol or placebo. This corresponds to inclusion rates of 36% among prescreened and 82% among fully screened patients. Nine participants failed the final screening, all due to pulse <60 bpm. Participant flow and exclusions are shown in Figure 2.

The baseline characteristics were well balanced between the two arms (Table 1). EAU high‐risk features were present in four placebo and six propranolol participants.

**TABLE 1:** Baseline characteristics of participants.

	Placebo^a^ (*n* = 20)	Propranolol^a^ (*n* = 20)	*p*‐value^b^
Age	66.0 (61.0, 71.0)	66.5 (63.5, 70.0)	0.7
PSA at baseline (μg/L)	8.4 (5.0, 12.5)	7.5 (6.6, 9.1)	0.6
Clinical tumour stage			0.3
1	9 (45)	12 (60)	
2	11 (55)	7 (35)	
3	0 (0)	1 (5.0)	
MRI tumour stage			0.2
2	12 (60)	16 (80)	
3a	7 (35)	2 (10)	
3b	0	1 (5)	
Missing^c^	1 (5)	1 (5)	
Prostate volume at MRI (mL)	41 (28, 45)	35 (25, 45)	0.5
Biopsy ISUP grade group			0.4
1	0 (0)	1 (5.0)	
2	15 (75)	9 (45)	
3	2 (10)	4 (20)	
4	2 (10)	3 (15)	
5	1 (5.0)	3 (15)	
ECOG Performance Status			>0.9
Fully active	20 (100)	20 (100)	
EAU risk group			0.7
Intermediate risk	16 (80)	14 (70)	
High risk	4 (20)	6 (30)	

Abbreviations: EAU, European Association of Urology; ECOG, Eastern Cooperative Oncology Group; ISUP, International Society of Urological Pathology; MRI, magnetic resonance imaging; PSA, prostate‐specific antigen.

^a^ Median (interquartile range); *n* (%).

^b^ Wilcoxon rank sum test for continuous variables; Fisher's exact test for categorical variables.

^c^ Counts of missing values (*n* =) are only displayed where data are incomplete.

### 3.2 Dosing, compliance and serum propranolol levels

Pre‐RALP compliance exceeded 98% in both groups (placebo 98.95% [SD 3.5]; propranolol 98.45% [SD 2.8]). Postoperative adherence was lower in the propranolol arm (placebo 98.45% [SD 2.8] vs. propranolol 86.03% [SD 28.1]). Dose‐escalation criteria were met, with subsequent increase to 40 mg twice daily in 13 placebo and eight propranolol participants; two participants in the propranolol group required dose reduction due to intolerance.

Serum propranolol (nmol/L) was undetectable at baseline in all participants (Figure S1). Preoperatively, no detectable levels were observed in the placebo group, whereas in the propranolol arm, 11 of 12 participants receiving 40 mg/day had detectable levels (median 79.8 nmol/L, interquartile range [IQR] 28.6–98.4) and seven of the eight receiving 80 mg/day had detectable levels (median 122.9 nmol/L, IQR 79.3–155.0); one participant in the 80 mg/day group was not sampled preoperatively. At the end of medication, or within a few days thereafter, detectable concentrations were present in 16 propranolol recipients (median 43.3 nmol/L, IQR 8.7–98.2), whereas none were detected in the placebo group.

### 3.3 BP, pulse rate, catecholamine metabolites and HADS measurements

**FIGURE 3:**
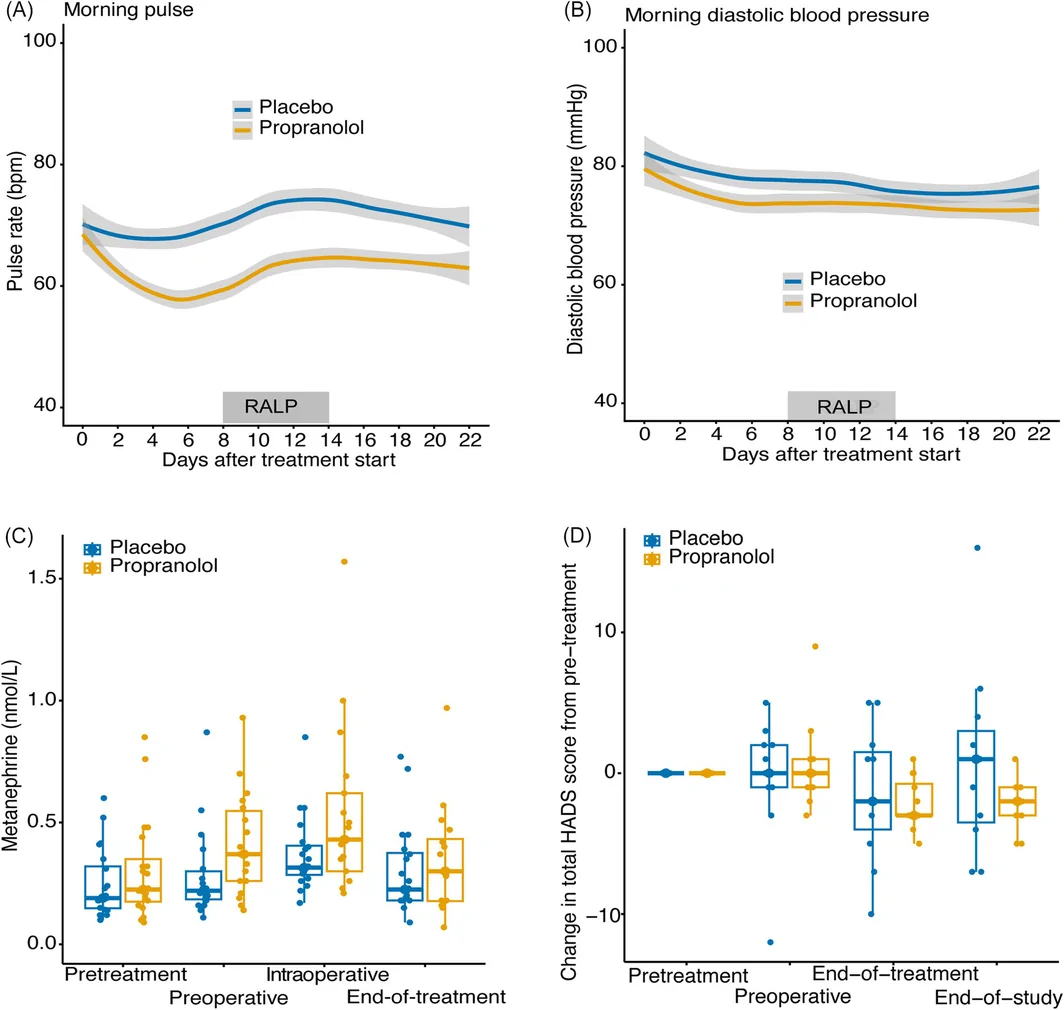
Blood pressure, pulse, catecholamine metabolites and Hospital Anxiety and Depression Scale (HADS) measurements. (A) Loess‐fitted plot of self‐monitored pulse changes from the start to the stop of treatment, differentiated by treatment arm; the grey area indicates the confidence interval. (B) Loess‐fitted plot of self‐monitored diastolic blood pressure changes from the start to the stop of treatment, differentiated by treatment arm; the grey area indicates the confidence interval. (C) Boxplot of metanephrine levels (nmol/L) in plasma by treatment group at four time points: pretreatment, preoperative, intraoperative and at the end of treatment. (D) Boxplot of changes from baseline in the anxiety component of the HADS stratified by treatment arm. RALP, robotic‐assisted laparoscopic prostatectomy.

Home monitoring demonstrated a marked reduction in pulse rate with propranolol (Figure 3A), most pronounced in the first week, with modest reductions in diastolic and systolic BP in both arms (Figures 3B and S2A). The epinephrine metabolite metanephrine showed a non‐significant increase following neoadjuvant propranolol treatment, whereas no change was observed in the placebo group (Figure 3C). Metanephrine and aldosterone levels increased intraoperatively in both groups (Figures 3C and S2B); these changes were transient and had nearly returned to baseline by the end of treatment. Normetanephrine, a norepinephrine metabolite, and cortisol levels remained stable in both arms, apart from a transient intraoperative decrease in cortisol in both groups due to oral dexamethasone administered immediately pre‐RALP as part of standard anaesthesiologic care (Figure S2C,D).

A complete set of four HADS questionnaires was filled out by 12 of 20 placebo participants and 11 of 20 propranolol participants (Table S3). In both treatment arms, the median total HADS score decreased from baseline to the end of treatment (Figure 3D). In the placebo group, this reduction was transient, with end‐of‐treatment changes ranging from −3.5 to 3.0 points (IQR). In contrast, in the propranolol group, the reduction remained below baseline, with an IQR of −3.0 to −1.0 points. HADS score values are shown in Figure S3A,B.

### 3.4 Perioperative parameters and TCD assessment

Anaesthesia and operative durations were comparable; the propranolol arm exhibited a mean anaesthesia duration that was 3.3% longer (~7 min) and an operative time that was 3.9% longer (~6 min) (Table 2). Estimated blood loss was 12% lower (33 mL) in the propranolol arm. Mean arterial pressure (MAP, mmHg) remained >70 at induction and at anastomosis in both arms, with lowest intraoperative MAP >60. Pulse rate was lower with propranolol at both time points; vasoactive medications, administered at the anaesthesiologist's discretion, were used more frequently in the propranolol group (85% vs. 60%) (Table S4).

**TABLE 2:** Perioperative parameter.

	Placebo^a^ (*n* = 20)	Propranolol^a^ (*n* = 20)	*p*‐value^b^ ^,^ ^c^
Duration (min)			
General anaesthesia	214 (37)	221 (51)	0.65
Surgical procedure	152 (36)	158 (49)	0.68
30° Trendelenburg position	144 (38)	150 (51)	0.63
Blood and fluid (mL)			
Blood loss	276 (173)	243 (160)	0.53
Missing	1	0	
IV fluids	1811 (613)	1912 (584)	0.6
Blood transfusions	None	None	
MAP (mmHg)			
Lowest MAP registered	62 (4)	62 (9)	0.95
MAP at induction anaesthesia	71 (13)	71 (12)	0.94
MAP at vesicourethral anastomoses	78 (20)	74 (27)	0.62
Missing	0	1	
Pulse rate (beats per minute)			
At induction of anaesthesia	65 (14)	53 (9)	0.004
At vesicourethral anastomoses	64 (19)	53 (20)	0.08
Missing	0	1	
TCD—Mean flow velocity middle cerebral artery (cm/s)			
At induction anaesthesia	36	40	*0.33*
At vesicourethral anastomosis	34	39	*0.12*
Missing	1	5	
Vasoactive medication			
Administered during procedure	12 (60)	17 (85)	0.18^c^

Abbreviations: MAP, mean arterial pressure; TCD, transcranial Doppler.

^a^ Mean (SD); *n* (%).

^b^ Welch two‐sample *T*‐test for continuous variables; Pearson's chi‐squared test for categorical variables.

^c^ Fisher's exact test; counts of missing values (*n* =) are only displayed where data are incomplete.

TCD was performed on 36/40 participants, of whom sufficient quality sonography was obtained in 32/40. Cerebral blood flow velocity was not reduced in the propranolol group compared to the placebo group.

### 3.5 Safety

No CTCAE v5.0 grade ≥3 AEs were attributed to propranolol, and no surgeries were delayed due to trial participation. Overall, 22/40 of participants reported AEs (9 receiving placebo and 13 receiving propranolol); three SAEs occurred, all in the placebo group (Table S5).

### 3.6 PSA

Mean PSA levels decreased more in the propranolol arm (−0.65 ng/mL) than in the placebo arm (−0.06 ng/mL). The interval between these measurements was similar in both arms: placebo, 7.9 days (SD 2.3); propranolol, 8.2 days (SD 2.2). One propranolol recipient had persistent PSA ≥ 0.1 ng/mL at the end of follow‐up, whereas there were none in the placebo group. Given the small sample size, these between‐group differences in PSA are exploratory and hypothesis‐generating only and cannot be interpreted as evidence of oncological efficacy.

## 4 DISCUSSION

PeP‐RALP, a randomised, double‐blind, placebo‐controlled pilot study, demonstrates the feasibility of administering propranolol perioperatively in men undergoing RALP for EAU intermediate‐ and high‐risk prostate cancer.

The prescreening‐to‐randomisation rate of 36% of RALP patients and the high preoperative adherence to study medication, without delays to surgery, indicate that perioperative propranolol can be reliably implemented within routine clinical RALP workflows. The decrease in postoperative adherence and reduced tolerance of the highest dose in the propranolol arm indicate a need for adjustments to dose‐escalation criteria and for strategies to improve compliance and tolerance in the postoperative period (e.g., enhanced patient information regarding postoperative fatigue).

### 4.1 Safety

As this study examined perioperative propranolol, we selected doses within the lower‐to‐mid range of commonly used clinical regimens (starting dose with escalation on Day 4). Intraoperative invasive BP monitoring was included to address safety concerns. Similar feasibility studies using comparable dosing regimens have reported no serious safety issues.^10^, ^11^, ^12^

The POISE randomised trial reported a higher incidence of stroke and overall mortality with perioperative β‐blockade^18^; however, POISE included less fit patients and used higher doses of a β_1_‐selective β‐blocker, whereas our trial enrolled fitter patients and used lower doses of a non‐selective β‐blocker.

In our study, no CTCAE v5.0 grade ≥3 AEs were attributed to propranolol, overall AEs were balanced between groups and no SAE occurred among propranolol recipients. These findings suggest that a full‐scale RCT is feasible from a safety perspective.

Potential cerebral risks associated with Trendelenburg position and pneumoperitoneum, as well as historical concerns that propranolol may impair intracranial autoregulation, were explored with TCD data. Usable data from 80% of participants showed no evidence of critical reductions in cerebral blood flow, supporting cerebral safety under typical RALP conditions and propranolol exposure.

For safety, we predefined eligibility and dose escalation to include only participants with pulse rate ≥60 bpm, anticipating pulse reduction as the main drug effect. Although the propranolol arm's mean pulse rate was <60 bpm, no participants experienced CTCAE v5.0 ≥ 3 bradycardia. The screening cut‐off pulse rate of ≥60 bpm appeared to exclude the fittest men; hence, a lower pulse threshold may be considered in future trials.

### 4.2 Perioperative challenges

Anaesthesia and surgery durations were similar in both groups, and mean intraoperative MAP was >60 across arms, consistent with safe use of propranolol during surgery. Importantly, no participants in the propranolol group experienced intraoperative hypotension or bradycardia unresponsive to standard treatment measures. The lower pulse rate in the propranolol arm likely contributed to more frequent use of vasoactive medication at the anaesthesiologist's discretion and supports prespecifying haemodynamic management protocols in future trials.

### 4.3 Stress hormones and psychological stress

The expected perioperative rise in metanephrines occurred in both treatment groups. The preoperative metanephrine increase observed in the propranolol group may reflect a compensation mechanism induced by decreased cardiac output, consistent with an early phase of neurohumoral adaptation following treatment initiation. This finding underscores the importance of an adequate preoperative exposure period, and future studies should therefore preserve at least the same interval of 1 week from propranolol initiation to RALP as applied in this pilot.

The exploratory HADS assessments were intended to quantify psychological stress and acceptability, but completion rates were suboptimal; although the available data do not suggest any major distress signal, we consider patient‐reported distress measures optional rather than essential for a larger efficacy study.

### 4.4 PSA changes

The preoperative PSA decline (~8 days) observed with propranolol should be considered hypothesis‐generating rather than evidence of efficacy, as associations between PSA changes and outcome are primarily based on longer term (annual) PSA velocity measures.^19^ Accordingly, for a future efficacy study, we propose BCR as the primary endpoint, given that both BCR and time to BCR are associated with adverse oncological outcomes.^20^

### 4.5 Clinical need for adjunct therapy to reduce recurrence rates after RALP

RALP is the main curative treatment for non‐metastatic prostate cancer, yet approximately 40% of EAU intermediate‐ and high‐risk patients develop BCR, underscoring the need for perioperative adjuncts.^21^, ^22^, ^23^, ^24^ Intensified neoadjuvant hormonal combinations (androgen deprivation therapy plus androgen‐receptor signalling inhibitors) have reduced BCR/residual disease in phase 2 studies but have not yet been established for routine clinical use pending confirmatory trials.^25^, ^26^ The phase 2 ARNEO study found no significant difference in BCR with degarelix ± apalutamide followed by RALP.^27^ A more definite answer should come from the multinational phase 3 PROTEUS trial, which will randomise ~2500 participants to 6 months of intensified hormonal combination versus androgen deprivation therapy and placebo, with metastasis‐free survival as a primary outcome.^28^ However, a limitation of these trials is the use of androgen deprivation therapy in both study arms.

Given that prostate cancer‐specific mortality after RALP is generally low, any adjuncts should prioritise safety and minimal toxicity; therefore, time‐limited, low‐toxicity perioperative strategies are particularly attractive.^29^, ^30^ In this context, alongside advances in preoperative surgical planning and optimisation for RALP,^31^ transient perioperative targeting of surgery‐induced catecholamine surges and downstream β_2_‐adrenergic signalling represents a biologically plausible, low‐cost approach that warrants definitive testing.

### 4.6 Recruitment and logistical considerations, and design optimisation for a future efficacy trial

PeP‐RALP was designed as a pilot study to evaluate the safety, tolerability and logistical integration of perioperative propranolol within RALP workflows in order to inform future efficacy trials. To this end, the study included secondary and exploratory endpoints, such as TCD, plasma catecholamines and serial home BP and pulse monitoring, to evaluate cerebral safety, stress hormone responses and haemodynamic stability. Based on the physiological and safety observations from this pilot, these monitoring procedures can be omitted in future efficacy studies. Consequently, a future multicentre trial building on our findings and powered for a clinical primary endpoint, such as BCR, will benefit from a pragmatic design with a simplified visit schedule.

Although the 36% prescreening‐to‐randomisation rate demonstrates overall feasibility, centre‐specific logistics directly constrained patient accrual: 17 prescreened patients (15% of all prescreened cases) were excluded because their single preoperative outpatient visit occurred <8 days prior to RALP, providing insufficient lead time for drug initiation. Additionally, all nine screen failures among fully screened patients were due to a baseline pulse <60. Future multicentre trials must account for such variable referral pathways to ensure steady accrual across participating centres.

To maximise accrual and scalability in future efficacy trials, we recommend four key design optimisations: (1) introduce trial participation at initial diagnosis to avoid surgical scheduling bottlenecks; (2) omit TCD, stress hormone assays and daily home BP and pulse monitoring, as this pilot established cerebral safety, expected intraoperative neurohumoral surges and haemodynamic stability; (3) allow a 7‐ to 21‐day preoperative dosing period to accommodate surgical scheduling while maintaining the 7‐day minimum exposure; and (4) lower the baseline pulse exclusion threshold to ≥55 bpm to prevent the unnecessary exclusion of physically fit men.

### 4.7 Exploratory sample‐size estimation for a future efficacy trial

Assuming conservative postoperative 5‐year BCR rates of approximately 30% in EAU intermediate‐ and high‐risk patients treated with RALP^23^, ^24^ and a hazard ratio of 0.75 (less extreme than the adjusted hazard ratio of 0.64 observed in our pharmaco‐epidemiologic registry study of incidental non‐selective β‐blocker use at radical prostatectomy^13^), we performed an exploratory sample‐size calculation for a future efficacy RCT. Using a two‐sided α=0.05, 80% power and 1:1 randomisation, this calculation indicates that approximately 1270 participants (about 380 BCR events at 5‐year follow‐up) would be required. Given the prescreening‐to‐inclusion rate of 36% observed in PeP‐RALP, this corresponds to screening approximately 3530 RALP patients. These estimates are post hoc and intended solely to guide the design and feasibility assessment of a definitive multicentre trial.

### 4.8 Limitations

As a pilot study, the sample size was designed to provide feasibility and parameter estimates rather than to detect group differences; therefore, the study was not powered to assess oncological efficacy, and no conclusions regarding oncological outcomes can be drawn.

## 5 CONCLUSIONS

This pilot RCT demonstrates the feasibility and perioperative safety of integrating propranolol into RALP pathways and supports a multicentre, adequately powered RCT that incorporates prespecified stratification by EAU risk group, standardised anaesthetic and haemodynamic management protocols and structured support to optimise postoperative adherence.

Primary oncological outcomes should include BCR, time to BCR and time‐to‐salvage therapy, whereas secondary endpoints may include validation of transcriptomic classifiers. Estimates of recruitment yield, adherence and safety derived from this pilot should inform sample size determination and operational planning.

## AUTHOR CONTRIBUTIONS

*Study concept and design*: Shivanthe Sivanesan, Kristin Austlid Taskén, Håkon Ramberg, Thomas Kristiansen, Suraj B. Thapa, Viktor Berge, PMT, Alfonso Urbanucci, Fredrik Ottosson, Anne M. H. Storås, Bjørn Brennhovd, Helene Hartvedt Grytli, Lars Bjerke. *Acquisition of data*: Shivanthe Sivanesan, Thomas Kristiansen, Marthe Bjøre, Sandra R. Dahl, Fredrik Ottosson, Bjørn Brennhovd. *Analysis and interpretation of data*: Shivanthe Sivanesan, Kristin Austlid Taskén, Håkon Ramberg, Thomas Kristiansen, Suraj B. Thapa, Viktor Berge, Per M. Thorsby, Sandra R. Dahl, Helene Hartvedt Grytli. *Statistical analysis*: Shivanthe Sivanesan, Kristin Austlid Taskén, Håkon Ramberg. *Drafting of the manuscript*: Shivanthe Sivanesan, Kristin Austlid Taskén, Håkon Ramberg. *Critical revision of the manuscript for important intellectual content*: All authors. *Obtaining funding*: Shivanthe Sivanesan, Kristin Austlid Taskén. *Administrative, technical, or material support*: Kristin Austlid Taskén, Thomas Kristiansen, Marthe Bjøre. *Supervision*: Kristin Austlid Taskén. *Final approval of the version to be published*: All authors.

## CONFLICT OF INTEREST STATEMENT

The authors declare no conflicts of interest.

## DISCLOSURE

During the preparation of this manuscript, ChatGPT (OpenAI, version 5.1) was used as an aid for language editing. The authors critically reviewed and edited the content and take full responsibility for the final manuscript.

## Supporting information


**Table S1.** Inclusion and Exclusion Criteria.
**Table S2**. Objective ‐Endpoints – Assessments.
**Table S3**. Completeness of HADS forms by treatment arm.
**Table S4**. Overview of perioperative vasoactive medication.
**Table S5**. Adverse Events.

**Figure S1.** Serum propranolol levels by treatment arm.

**Figure S2.** A‐D Systolic blood pressures, normetanephrine‐, Cortisole‐ and Aldosterone‐ levels.

**Figure S3.** A,B. HADS‐Anxiety and HADS‐Depression scores.

## Data Availability

The data that support the findings of this study are available from the corresponding author upon reasonable request.
