# Supplementary material for: Perioperative propranolol during robotic‐assisted radical prostatectomy: A randomised, double‐blind, placebo‐controlled, pilot trial (PeP‐RALP)
Source: BJUI Compass. 2026 Aug 26;7(9):e70267. doi: 10.1002/bco2.70267 (PMC13519088; doi:10.1002/bco2.70267)
Supplement: Supplementary file 1 [file BCO2-7-e70267-s003.docx]

Supplementary material

[Table S1 - Inclusion and Exclusion Criteria	1](#_Toc227858963)

[Table S2 Objective -Endpoints – Assessments	3](#_Toc227858964)

[Table S3 – Completeness of HADS forms by treatment arm	5](#_Toc227858965)

[Table S4 – Overview of perioperative vasoactive medication	6](#_Toc227858966)

[Table S5 -Adverse Events	7](#_Toc227858967)

[Figure S1. Serum propranolol levels by treatment arm	8](#_Toc227858968)

[Figure S2A-D Systolic blood pressures, normetanephrine- , Cortisole- and Aldosterone- levels	8](#_Toc227858969)

[Figure S3A,B. HADS-Anxiety and HADS-Depression scores	8](#_Toc227858970)

[Protocol Amendment history:	9](#_Toc227858971)

[Schedule of Activities	13](#_Toc227858972)

### Table S1. Inclusion and Exclusion Criteria

**Inclusion criteria:**

**Age**

Participant must be 40 to 80 years of age at the time of signing the informed consent.

**Type of Participant and Disease Characteristics**

Participants with PCa stratified into EAU IR and HR for BCR planned for curative RALP Participants who are in the ECOG PS 0-1 as determined by medical evaluation including medical history, physical examination, laboratory tests, electrocardiogram (ECG).

**Informed Consent to PeP-RALP study**

1. Willing to-, and capable of, giving signed informed consent in Norwegian (as described in Appendix 1) which includes compliance with the requirements and restrictions listed in the informed consent form (ICF) and in this protocol.

**Exclusion criteria:**

**Medical Conditions**

1. Sick sinus syndrome
2. Atrioventricular (AV) block grade 2 and 3
3. Recent (3 months) myocardial infarction
4. Known unstable- or vasospastic- angina
5. Heart failure (New York Heart Association [NYHA] > 2)
6. Symptomatic peripheral vascular disease (e.g. intermittent claudication)
7. Known pulmonary hypertension
8. Known carotid artery stenosis or recent (3 months) stroke
9. Bronchial asthma or other chronic obstructive pulmonary disease (COPD)
10. Kidney failure (estimated Glomerular filtration rate [eGFR]<50)
11. Liver failure (cirrhosis, jaundice, signs of hepatic decompression)
12. Unregulated diabetes mellitus
13. Untreated thyroid disorder
14. Depressive episode within last 6 months (within last 12 months if major depressive episode)
15. Known drug allergy against propranolol or excipients
16. Any medical conditions considered to prohibit Propranolol use as judged by the treating physician (including frailty).
17. Participants with known substance- or alcohol-abuse

**Prior/Concomitant Therapy**

18. Recent (<3 month) use of systemic beta-blockers prior to screening.
19. Patients receiving non-dihydropyridine calcium channel blocking agents (eg diltiazem, verapamil)
20. Patients receiving anti-arrhythmic agents (e.g. amiodarone, sotalol, digoxin, verapamil, flecainide)
21. Patients receiving digoxin, rizatriptan, hydralazine, fluvoksamin, or fluoksetin
22. Patients using daily anxiolytics (e.g. benzodiazepines), alpha-receptor adrenergic agonists (e.g. clonidine)
23. Recommendations in the Summary of Product Characteristics for propranolol regarding concomitant use of other medications will be adhered to

**Diagnostic assessments**

24. Sinus bradycardia (pulse rate <60 beats/minute)
25. Resting blood pressure <110/60mmHg OR >160/100
26. AV-block grade 2 and 3 on ECG

### Table S2. Objectives, Endpoints, and Assessments

| **Objective** | **Endpoints** | **Assessment** |
| --- | --- | --- |
| **Primary:** |  |  |
| Feasibility of conducting a formal larger RCT to compare the efficacy of propranolol vs placebo to decrease PCa recurrence following RALP. | Numbers needed to screen  Compliance before RALP >80%  Compliance after RALP > 80% | Number of patients needed to screen to include 40 patients.  Compliance to medication. |
| **Secondary:** |  |  |
| Safety and tolerability of PeP-RALP intervention | Proportion of patients experiencing treatment related clinical significant hypotension and/or bradycardia.  Adverse events of PeP-RALP medication.  Proportion of patients tolerating daily dose of 80mg propranolol. | Home monitoring of blood pressure and pulse rate twice daily  CTCAE v5.0  Proportion of patients completing the 80 mg propranolol interval |
| Determine the effect of RALP on catecholamine levels | Changes in catecholamine levels in the perioperative period. | Plasma levels of metanephrine, normetanephrine and metoxytyamine  1. Baseline (pre PeP-RALP medication)  2. Pre- intra- and post- RALP  3. At end of PeP-RALP medication |
| Determine the bioavailability of propranolol | Serum levels of propranolol pre-operatively and at end of PeP-RALP medication. | Quantification of propranolol using |
| Determine the effect of preoperative propranolol treatment on the serum level of  PSA | Changes in PSA levels after 7-14 days of PeP-RALP medication. | Serum PSA immunoassay |
| To determine the effect of propranolol on post-operative biochemical failure | Proportion of patients with serum PSA levels above 0.1 ng/ml at 6 weeks post-RALP. | Serum PSA immunoassay |
| Surgical complications in PeP RALP patients | Frequency and severity of surgical complications | Clavian-Dindo classification |
| Intraoperative challenges | Anesthesiological challenges.  Surgical challenges. | Intraoperative MAP <60 mm/Hg  Need of vasopressors (dose, times administered).  Surgical procedure time.  Estimated intraoperative blood loss. |
| **Exporatory:** |  |  |
| Investigate alterations in perioperative perceived distress | Change in perceived distress during the study. | HADS questionnaire |
| Determine alterations in immune activities in response to pre-operative propranolol treatment | Difference in systemic and intra-tumor immune cell infiltration and activity in response to propranolol between treatment groups. | Immunohistochemistry and image mass cytometry to address intra-tumoral and ELISA and flow cytometry to address systemic effects. |
| Determine the effect of pre-operative propranolol treatment on prognostic markers and assess for predictive biomarkers | Difference in prognostic markers between treatment groups  Identify predictive biomarkers for propranolol responsiveness | Decipher GRID transcriptome analyses (e.g. PAM50 and Decipher Prostate Cancer Classifier) |
| Intraoperative alterations in cerebral autoregulation and intracranial pressure | Differences between propranolol and placebo with regard to cerebral blood flow characteristics | Transcranial Doppler flow velocity measurement of middle cerebral artery |
| RCT =Randomized Controlled Trial; PCa = Prostate cancer; RALP = Robotic assisted laparoscopic prostatectomy; CTCAE = Common Terminology Criteria for Adverse Events; PSA = Prostate specific antigen | | |

### Table S3. Completeness of HADS forms by treatment arm

|  | **Propranolol** | | **Placebo** | | |
| --- | --- | --- | --- | --- | --- |
| **HADS questionnaire filled out by pariticipants** | | | | | |
| HADS no. 1 | 19 | 95 % | 18 | 90 % |  |
| HADS no. 2 | 16 | 80 % | 16 | 80 % |  |
| HADS no. 3 | 15 | 75 % | 15 | 75 % |  |
| HADS no. 4 | 17 | 85 % | 15 | 75 % |  |
| **Combinations of HADS questionnaires filled out by participants** | | | | |  |
| HADS no. 1,2 | 16 | 80 % | 14 | 70 % |  |
| HADS no 1,2,3 | 14 | 70 % | 14 | 70 % |  |
| HAD1,2,3,4 | 11 | 55 % | 12 | 60 % |  |

**Table S3. Completeness of HADS forms by treatment arm**

HADS questionnaire was filled out by the participants at four timepoints; around time of inclusion (HADS1), surgery (HAD2), at end-of-treatment (HAD3) and at end-of-study (HAD4). An electronic capture solution was in place at study start, during the study a paper based back-us solution was used to increase the answer rates.

### Table S4. Overview of perioperative vasoactive medication

|  | **Propranolol^1^**  (n = 20) | **Placebo^1^**  (n = 20) | **p-value***^2^* |
| --- | --- | --- | --- |
| Vasoactive medication administered | 17(85) | 12(60) | 0.155 |
| Type of vasoactive medication: |  |  |  |
| Ephedrine | 9(45) | 9(45) | 1.000 |
| Ephedrine and Atropine | 4(20) | 0 | 0.106 |
| Ephedrine and Phenylephrine | 1(5) | 1(5) | 1.000 |
| Ephedrine, Atropine and Phenylephrine | 1(5) | 0 | 1.000 |
| Ephedrine, Atropine, Phenylephrine and Noradrenaline | 2(10) | 0 | 0.487 |
| Phenylephrine | 0(0.0) | 2(10.0) | 0.487 |
| **Dose of vasoactive medication (mg):** |  |  |  |
| Ephedrine | 15.00 [10.00–27.50] | 10.00 [5.00–15.00] | 0.085 |
| Atropine | 0.60 [0.60–0.80] |  | NA |
| Phenylephrine | 0.10 [0.10–0.15] | 0.40 [0.20–2.19] | 0.064 |
| Noradrenaline | 0.07 [0.07–0.07] | NA | NA |
| ^1^ N(%) mg[IQR], ^2^ Fishers exact test for categorical variables, Mann-Whitney-U test for continuous variables | | | |

**Table S4. Overview of perioperative vasoactive medication**

Overview of vasoactive medication administered perioperatively, and total dose of vasoactive medication administered, stratified per treatment arm.

### Table S5. Adverse Events

|  | **ID** | **CTCAE v5.0 grade** | **Adverse event** |
| --- | --- | --- | --- |
| **Placebo** | 2 | 2 | Urinary tract infection |
|  | 6 | 3 | Infected heamatoma |
|  | 9 | 1 | Dizziness |
|  | 10 | 1 | Headache with epistaxis |
|  | 15 | 1 | Dizziness postoperative |
|  | 33 | 2 | Urinary tract infection |
|  | 35 | 1 | COVID-19 |
|  | 43 | 1 | Fever throat pain |
|  | 50 | 3 | Cholecystitis |
| **Propranolol** | 1 | 1 | Dysuria cold fingers |
|  | 3 | 2 | Musckle wakness (spinal stenosis) |
|  | 7 | 1 | Heamaturia genital swelling |
|  | 8 | 1 | Abdominal cramps |
|  | 12 | 1 | Very fatigued and dizzy post op |
|  | 14 | Not graded | Slight fatigue post op |
|  | 16 | 1; 2 | Nausea fatigue (day 4-6); Urinary tract infection |
|  | 18 | 1 | Lightheaded (day 9 -10) |
|  | 20 | 1 | Headache |
|  | 24 | 1 | Eczema worsened |
|  | 27 | 1 | Asymptomatic bradykardia |
|  | 28 | 1 | Common flu |
|  | 29 | 1 | Urinary tract infection |
|  | 38 | 1 | Decreased sensibility left foot, recovered before end of study |
|  | 46 | 1; 1 | COVID-19; decreased sensation left fingers |
|  | 49 | 1 | Nausea |
| ID = Unique anonymized participant number; CTCAE = Common Terminology Criteria  for Adverse Events; UTI =Urinary tract infection | | | |

**Table S5 . Adverse Events**

Table with all participants with one or more recorded adverse events with corresponding CTCAE v5.0 grade, stratified by treatment arm.

### Figure S1. Serum propranolol levels by treatment arm

**Figure S1. Serum propranolol levels by treatment arm**

Boxplots of serum propranolol concentrations (nmol/L) by treatment group at three time points: pretreatment, preoperative, and end of treatment. In the propranolol arm, 2/20 participants had non‑detectable preoperative serum propranolol; one due to missing sampling and possibly due to low compliance for 1–2 days preoperatively. At end of treatment, some samples were obtained a few days after the last study dose because visits occasionally fell on weekends, which may have contributed to lower measured concentrations

### Figure S2A-D. Systolic blood pressures, and levels of; Aldosterone, Nometanephrine and Cortisol

**S2A-D. Systolic blood pressures, and levels of; Aldosterone, Nometanephrine and Cortisol
A:** Loess-fitted plot of systolic blood pressure changes from start to stop of treatment, differentiated by treatment arm. Grey area indicates the confidence interval
**B:** Boxplot of Aldosterone levels (pmol/L) in plasma by treatment group at four time-points; pre-treatment, preoperative, intraoperative, at end-of-treatment.
**C:** Boxplot of normetanephrine levels (nmol/L) in plasma by treatment group at four time-points; pre-treatment, preoperative, intraoperative, at end-of-treatment.
**D:** Boxplot of Cortisol levels (pmol/L) in serum by treatment group at four time-points; pre-treatment, preoperative, intraoperative, at end-of-treatment.

### Figure S3A,B. HADS-Anxiety and HADS-Depression scores

**Supplementary Figure 3A,B. HADS-Anxiety and HADS-Depression score values**

**A:** Scatterplot of HADS-Anxiety scores at four timepoints, stratified by treatment arm. **B:** Scatterplot of HADS-Depression scores. A score of 0-7 is considered normal, 8-10 borderline abnormal, 11-21 abnormal

### Protocol Amendment history:

Amendment 2: PeP-RALP v2.0 (29.11.2022)

| **Section # and Name** | **Description of Change** | **Brief Rationale** |
| --- | --- | --- |
| 5.1. Inclusion Criteria | Removed that participant has to consent to Forskningsbiobank for Kreft | Input from Regional Ethics Committee |
| 11.2.2. IMPD | Error in amount of capsules per box changed from 48 capsules – 100 capsules | Error in the sum of capsules needed per participant, no change in dosage |
| Throughout | Minor editorial and document formatting revisions | Minor, therefore have not been summarized |

Amendment 3: PeP-RALP v2.1 (12.12.2022)

| **Section # and Name** | **Description of Change** | **Brief Rationale** |
| --- | --- | --- |
| Throughout | Minor editorial and document formatting revisions | Minor, therefore have not been summarized |

Amendment 4: PeP-RALP v2.2 (16.01.2023)

| **Section # and Name** | **Description of Change** | **Brief Rationale** |
| --- | --- | --- |
| 1.3. Schedule of Activities | Screening window is changed to allow screening until the day of treatment start (Day 1). Previously screening was allowed until the day before treatment start (Day -1). | To avoid to burden the participant with an extra screening visit in cases where the patient is scheduled for a hospital appointment in the morning of Day 1. |
| 1.3. Schedule of Activities | The visit widows are edited from the format “±” X days to “+” X days e.g. visit widow for RALP previously displayed as 11 ±3 days, now displayed as 8 +6 days. | To make the Schedule of Activities easier to interpret. |

Amendment 5: PeP-RALP v3.0 (19.03.23)

| **Section # and Name** | **Description of Change** | **Brief Rationale** |
| --- | --- | --- |
| 5.3 Exclusion criteria | Exclution criterion no. 26 is speciefied to AV block grade 2 and 3 on ECG. . | Errata. As seen in exclusion criteria 2. Only patients with AV block ≥2 are to be excluded |
| 6.7 Concomitant therapy | Subdivision of concomitant medication into   - 6.7.1. Out patient concomitant medication from time of enrollment to end of study: - 6.7.2. In patient concomitant medication received at the hospitalization for RALP   Registration of daily dosage administered is sufficient. | During hospitalization for RALP all medication administered to is registered in the electronic patient chart – Metavsion)  More 10 than standard medications are administered during the short RALP hospitalization. Recording all of these medications in the eCRF is not necessary. Table 6.7.2 specify which medications to be registered.  (Standard medication administered during the RALP hospitalization includes, mild and strong opioid analgesics, steroids, Cox 2 inhibitors, paracetamol, local and general anesthetics, antiemetics, vasopressors and anticholinergic medication.) |
| 7.1 Discontinuation of Study Intervention | Editorial changes | To clarify that discontinuation of Study intervention is indicated with an SAE related to the study intervention, and not an SAE related to the RALP procedure. |
| 8.7 Adverse events of Special interest | Generally: CTCAEv 5.0 ≥3: Hypotension or sinus bradycardia  *During anesthesia:*   - *Intra operative hypotension (MAP <60) not responding to treatment* - *Intra operative bradycardia during surgery not responding to treatment* | Bradycardia and hypotension are common AE of propranolol. It is only of special interest if this is symptomatic and in need of intervention.  During anesthesia patients often become bradycardic and hypotension in response to the general anesthesia and surgical procedure. It is only of interest to record cases where hypotension/bradycardia do not respond to treatment measures. |
| 9.2.2. Vital signs | Repiratory rate may be obmitted if pulse oximetry is performed | Sufficient information about the patients is obtained by either an pulse oximetry or respiratory rate |

### Schedule of Activities

| **Activities** | **Screening** | **Pre-treatment** | **Randomization** | **Treatment Period** | | | | | | | | | | | | **Follow-up** | |
| --- | --- | --- | --- | --- | --- | --- | --- | --- | --- | --- | --- | --- | --- | --- | --- | --- | --- |
|  |  |  |  | **start** |  |  |  |  |  |  | **RALP** |  |  | **stop** |  | |  |
|  |  |  |  | **40 mg** | | | **80 mg** | | | | | | **40 mg** | | |  | |
| Visits | 1^1^ | 2^2^ | 2^2^ |  |  | 3^1^ |  |  |  | 4^1^ | 5^2^ | 6^1^ |  | 7^2^ | 8^1^ | |  |
| Days (from treatment start) | <-40 to + 1 | 1 | 1 | 1 | 2 | 3 | 4 | 5 | 6 | 7 | 8 | 9-19 | 20-21 | 22 | 49 | |  |
| Visit window (days) |  | - 7 | - 7 |  |  |  |  |  |  | + 6 | + 6 | + 6 | + 6 | + 6 | + 14 | |  |
| Informed consent | X |  |  |  |  |  |  |  |  |  |  |  |  |  |  | |  |
| Inclusion/exclusion criteria | X | X |  |  |  |  |  |  |  |  |  |  |  |  |  | |  |
| ECOG PS | X | X |  |  |  |  |  |  |  |  |  |  |  |  |  | |  |
| Demographics | X |  |  |  |  |  |  |  |  |  |  |  |  |  |  | |  |
| Medical history^3^ | X | X |  |  |  |  |  |  |  |  |  |  |  |  |  | |  |
| Physical examination |  | X |  |  |  |  |  |  |  |  |  |  |  |  |  | |  |
| Prior and concomitant medication | X | X |  | X | X | X | X | X | X | X | X | X | X | X | X | |  |
| Routine blood sample collection |  | X |  |  |  |  |  |  |  | X | (X^4^) |  |  |  | X | |  |
| Blood sample collection in co-operation with Forskningsbiobank for kreft OUS |  | X |  |  |  |  |  |  |  | X^5^ | (X^5^) X^6^ |  |  | X |  | |  |
| Serum PSA | X^7^ | X^7^ |  |  |  |  |  |  |  | X^8^ | ( X^8^) |  |  |  | X^7^ | |  |
| Tissue sample from Forskningsbiobank for kreft^9^. |  |  |  |  |  |  |  |  |  |  | X |  |  |  |  | |  |
| 12-lead ECG |  | X |  |  |  |  |  |  |  |  |  |  |  |  |  | |  |
| PROM –HADS |  | X |  |  |  |  |  |  |  | X |  |  |  | X | X | |  |
| Randomization |  |  | X |  |  |  |  |  |  |  |  |  |  |  |  | |  |
| Start treatment |  |  |  | X |  |  |  |  |  |  |  |  |  |  |  | |  |
| Physical examination |  | X |  |  |  |  |  |  |  |  |  |  |  |  |  | |  |
| Vital signs |  | X |  | X | X | X | X | X | X | X | X | X | X | X |  | |  |
| Intraoperative TCD measurements |  |  |  |  |  |  |  |  |  |  | X |  |  |  |  | |  |
| Intraoperative anesthesiological and surgical challenges |  |  |  |  |  |  |  |  |  |  | X |  |  |  |  | |  |
| Surgical complication |  |  |  |  |  |  |  |  |  |  |  |  |  |  | X | |  |
| Drug compliance |  |  |  | X | X | X | X | X | X | X | X | X | X | X |  | |  |
| Intraoperative anesthesiological and surgical challenges |  |  |  |  |  |  |  |  |  |  | X |  |  |  |  | |  |
| Assess AEs |  |  |  | X | X | X | X | X | X | X | X | X | X | X | X | |  |
| Assess SAEs |  |  |  | X | X | X | X | X | X | X | X | X | X | X | X | |  |

Abbreviations: RALP, Robotic Assisted Laparoscopic Prostatectomy; ECG, Electrocardiogram; PROM, Patient Reported Outcome Measures; HAD, Hospital Anxiety and Depression Scale; TCD, Transcranial Doppler; AE, Adverse Effects

**^1^** Visit includes phone visits or physical visits as suitable;

^2^Physical visit at hospital

^3^ Medical history includes substance usage (drugs, alcohol, tobacco),

^4^ The second routine (part of standard pre-operative clinical work-up) blood test is drawn either the day before RALP or at the day of RALP.

^5^ The second study specific (pre-operative) blood test is RALP drawn together with routine clinical work-up

^6^ Study specific blood sample drawn intra-operatively (via arterial blood line, see section 2.3.2 ) and/or in the recovery department immediately post operatively

^7^ PSA value from participants hospital patient record, i.e. in hospital values as well as external (e.g. office urologist, general practitioners)

^8^ Blood sampling for PSA measurement in co-operation with Forskningsbiobank for kreft OUS (see section 9.1.3.)if participant consent to Forskningsbiobank for kreft OUS

^9^ If participant consent to Forskningsbiobank for kreft OUS
